# Is HRQOL in dialysis associated with patient survival or graft function after kidney transplantation?

**DOI:** 10.1186/s12882-016-0316-5

**Published:** 2016-07-26

**Authors:** Nanna von der Lippe, Bård Waldum-Grevbo, Anna Varberg Reisæter, Ingrid Os

**Affiliations:** 1 Institute of Clinical Medicine, University of Oslo, Oslo, Norway; 2 Department of Nephrology, Oslo University Hospital, Oslo, Norway; 3 Department of Transplantation Medicine, Oslo University Hospital, Oslo, Norway; 4 The Norwegian Renal Registry, Oslo, Norway

## Abstract

**Background:**

Health related quality of life (HRQOL) is patient-reported, and an important treatment outcome for patients undergoing renal replacement therapy. Whether HRQOL in dialysis can affect mortality or graft survival after renal transplantation (RTX) is not determined. The aims of the present study were to investigate whether pretransplant HRQOL is associated with post-RTX patient survival or graft function, and to assess whether improvement in HRQOL from dialysis to RTX is associated with patient survival.

**Methods:**

In a longitudinal prospective study, HRQOL was measured in 142 prevalent dialysis patients (67 % males, mean age 51 ± 15.5 years) who subsequent underwent renal transplantation. HRQOL could be repeated in 110 transplant patients 41 (IQR 34–51) months after RTX using the self-administered Kidney Disease and Quality of Life Short Form (KDQOL-SF) measure. Kaplan-Meier plots were utilized for survival analyses, and linear regression models were used to address HRQOL and effect on graft function.

**Results:**

Follow-up time was 102 (IQR 97–108) months after RTX. Survival after RTX was higher in patients who perceived good physical function (PF) in dialysis compared to patients with poorer PF (*p* = 0.019). Low scores in the domain mental health measured in dialysis was associated with accelerated decline in graft function (*p* = 0.048). Improvements in the kidney-specific domains “symptoms” and “effect of kidney disease” in the trajectory from dialysis to RTX were associated with a survival benefit (*p* = 0.007 and *p* = 0.02, respectively).

**Conclusion:**

HRQOL measured in dialysis patients was associated with survival and graft function after RTX. These findings may be useful in clinical pretransplant evaluations. Improvements in some of the kidney-specific HRQOL domains from dialysis to RTX were associated with lower mortality. Prospective and interventional studies are warranted.

## Background

Patients with end stage renal disease (ESRD) may be treated with dialysis or renal transplantation (RTX). RTX is considered superior to chronic dialysis, as it prolongs survival and alleviates uremic symptoms [1, 2]. It is also considered more cost-effective based on quality adjusted life years [3]. Dialysis patients in Norway are considered with regard to eligibility for RTX, independent of chronological age. During the last decades, short-term graft and patient survival have improved due to upgraded surgical procedures, more efficient immunosuppressive regimens and improved care for complications and comorbidities. However, long-term term attrition rates for renal grafts have only shown small improvements, and survival in RTX patients is reduced compared to the general population [4–6].

Patient-related outcome measures (PROMs) have become important as they may capture the impact of disease perceived by the patients themselves. PROMs in a clinical setting are important for both the patient and the health provider as they may lead to more adequate intervention and treatment. The concept health-related quality of life (HRQOL) is an example of a PROM, and includes the subjective perception of e.g. physical function, mental well-being and social aspects.

Although aspects of HRQOL may improve after RTX compared with dialysis [7, 8], generic HRQOL is reported inferior by transplanted patients compared to that of the general population [1, 9]. Disease-specific HRQOL instruments may reveal other aspects of clinical importance than the generic tools, and unmask more subtle changes. Our group has recently reported that kidney-specific domains closely related to daily life complaints and nuisances improved the most after RTX [9].

HRQOL has been shown to be a predictor of morbidity and mortality in dialysis patients, also after multiple adjustments [10–12]. In a large, prospective study by Molnar Varga et al. [13], poor HRQOL measured after RTX was associated with mortality and graft failure. These observations support that PROMS are important in the follow-up of RTX patients.

Whether self-perceived physical function reported during dialysis may be associated with mortality after renal transplantation has recently been addressed in two trials [14, 15]. Both reported that pretransplant physical function was associated to survival after RTX. Whether other aspects of HRQOL measured in dialysis could predict mortality after RTX has not been addressed.

Deteriorating renal graft function is associated with immunologic and non-immunologic factors, including reduced HRQOL measured after RTX [16–18]. Whether poor HRQOL during the time of dialysis may be related to graft function has not previously been addressed. Nor do we know whether improvement in HRQOL in the transition from dialysis to transplantation may be associated with survival.

We hypothesized that poor HRQOL during dialysis could be related to mortality and reduced graft function after RTX, and that patients with improvement in HRQOL from dialysis to transplantation had better survival compared to patients without improvement. Thus, the aims of our study were to explore whether generic or kidney-specific HRQOL measured during chronic dialysis was associated with mortality or graft function after successful renal transplantation. Secondly, to assess whether improved self-perceived HRQOL in the transition from dialysis to transplantation could be related to patient survival.

## Methods

Between August 2005 and February 2007, a total of 301 prevalent dialysis patients from 10 different hospitals in Norway were included in a cross-sectional study addressing HRQOL issues. The study details have been described previously, and are therefore only briefly presented here [19]. All patients >18 years receiving either hemodialysis (HD) or peritoneal dialysis (PD) for more than two months were invited to participate. Patients came from both rural and urban areas. Cognitive dysfunction, major psychiatric disorder and inadequate Norwegian language skills were exclusion criteria. Hospitalization during the investigation period led to exclusion, but patients could be enrolled four weeks or more after hospital discharge if they were clinically stable. The questionnaires were answered by the patients during a regular HD session or during a scheduled visit for the PD patients.

In 2010–2011, a follow-up study was conducted, where patients who had taken part in the first study were invited to participate, whether they had undergone renal transplantation or were still in dialysis. Inclusion and exclusion criteria were unchanged from the previous study, except for the dialysis criteria. Transplanted patients completed the questionnaires during a regular visit at the renal outpatient clinic [9]. The present study provides results from transplanted patients only.

Clinical and demographical data were collected from hospital charts and/or direct questioning of the patients. Renal transplantation, graft function, patient survival, and cause of death were reported by the Norwegian Renal Registry [20]. Estimated glomerular filtration rate (eGFR) was calculated on the basis of the simplified Modification of Diet in Renal Disease prediction equation [21]. Graft function was defined as eGFR. Comorbidity was measured using the modified Charlson comorbidity index (CCI) [22, 23]. CCI has been validated for dialysis patients [23] and kidney transplanted patients [24], and is a composite score of age and 19 weighted comorbid conditions, including coronary artery disease, congestive heart failure, cerebrovascular disease, diabetes, malignancy and chronic pulmonary disease. Diabetes as a comorbid condition scored one point, and diabetes as cause of ESRD scored two points. In the present study, CCI was also calculated without including age in order to enable evaluation of age as a separate variable in multivariate analysis.

## HRQOL

The self-administered Kidney Disease and Quality of Life Short Form measure version 1.3 (KDQOL-SF) [25] consists of a kidney-specific and a generic part. The kidney-specific part contains 43 kidney-specific items that are summarized into 11 domains, i.e. symptoms, effect of kidney disease, burden of kidney disease, cognitive function, quality of social interaction, sleep, sexual function, social support, dialysis staff encouragement, patient satisfaction with care and work status. The generic part of KDQOL-SF comprises the Medical Outcome Study 36-item Short Form Health Survey (SF-36) [26]. It consists of 36 items summarized into the eight conceptual domains physical function, limitation due to physical problems, bodily pain, general health, vitality, social function, limitation due to emotional problems, and mental health. Scores in KDQOL-SF were transformed into linear 0–100 point scores, with higher scores indicating better quality of life. KDQOL-SF has been validated for kidney transplanted patients and dialysis patients [27, 28]. In the present study, the item concerning dialysis access was not included in the KDQOL domain “symptoms”, as it is irrelevant for transplanted patients.

Half a standard deviation (SD) of the baseline score in each HRQOL domain was chosen as a measure of clinical relevant change in HRQOL from dialysis to RTX [29]. This is equivalent to Cohen’s d effect size of = 0.50 [30]. According Cohen’s d, an effect size of 0.2–0.5 is regarded a small effect, 0.5–0.8 a medium effect and 0.8 to infinity a large effect.

## Statistics

Clinical and demographic data were presented as mean with SD if assumptions of normality were fulfilled, or as median with interquartile range (IQR) if data were skewed. Percentages were given for categorical variables. Dependent on the distribution of the variables, Student’s *t*- test or Mann–Whitney *U* test were used for comparison between two groups. Chi-square test was used to compare categorical variables. The observation period for the survival analyses was defined as time from transplantation until death or study end (May 2015).

Kaplan-Meier curves and log-rank statistics were applied to identify significant differences in survival. Death (including death after return to dialysis) was defined as end point. All the KDQOL-SF domains were investigated regarding survival except “dialysis staff encouragement” and “satisfaction with care”, as these domains were considered not relevant in transplanted patients. Based on pretransplant HRQOL scores, patients were categorized by tertile levels, and survival differences between the three patient groups were assessed.

Survival was compared between patients with clinical relevant improvement and patients with no clinical relevant improvement in the transition from dialysis to transplantation. The product term of age and HRQOL domains was entered in Cox regression analyses to check for interaction regarding survival.

Multiple linear regression analyses were performed with yearly eGFR decline as dependent variable. 1-year post-transplant eGFR was used as the reference point for calculating yearly decline, as eGFR levels might fluctuate during the first months after transplantation. For patients with graft loss, eGFR was set as zero the year they returned to dialysis. Patients who lost their graft or died within one year after transplantation were excluded in the regression analyses. Independent variables were age, gender, HRQOL domains, and comorbidity (CCI score without including age to avoid double adjustment for age). These variables were chosen on a clinical basis. The assumptions of linearity of continuous variables were checked and found to be adequately met.

Missing values in the generic part of KDQOL-SF were substituted with the patient’s mean score if less than half of the items were missing. No substitution was done in the kidney-specific part, if a question was left unanswered, the total score was calculated as suggested by the RAND group [25]. Missing data were treated by pairwise deletion in the statistical analyses.

All data were analysed using SPSS for Windows version 22 (IBM SPSS Statistics, New York, USA). Level of significance was set to *p* < 0.05.

## Results

### HRQOL measured in dialysis and survival

**Fig. 1:**
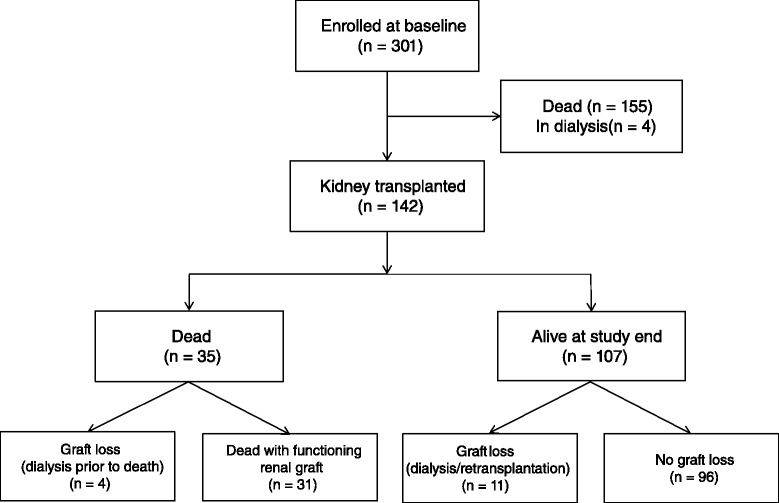
Flowchart of enrolment

Of the 301 prevalent dialysis patients who had completed the HRQOL questionnaires in 2005–2007, 142 were subsequently transplanted (Fig. 1). Clinical and demographic characteristics are given in Table 1. The immunosuppressive regimen of cyclosporine/mycophenolate mofetil/prednisolone was used in 43 %, while 39 % used a combination of tacrolimus/mycophenolate mofetil/prednisolone. Median time from baseline to RTX was 11 (range 0–64) months, time from RTX to death or study end was 7.4 (0.8–9.7) years.

**Table 1:** Demographic and clinical characteristics of patients in dialysis (*n* = 142) who were subsequently renal transplanted. During follow up, 35 patients died

I	Alive *n* = 107	Deceased *n* = 35	*p*-value
Age	46.9 ± 14.5	63.6 ± 10.9	< 0.001
Gender, male %	63.5	78.8	0.10
Time of follow up, months	106 (101–111)	76 (64–85)	< 0.001
Dialysis vintage, months ^ǂ^	7 (4–15)	9 (3–23)	0.32
Peritoneal dialysis, %	23	15	0.31
Waitlisted for RTX, %	68	60	0.40
Living donor, %	29	17	0.17
Hypertension, %	89	85	0.58
Cardiovascular disease, %	11	31	0.005
Charlson comorbidity index	4 (2–6)	6 (5–7)	0.001
Diabetes, %	23	15	0.32
Body mass index, kg/m^2^	25.4 ± 4.4	25.7 ± 4.2	0.52
Systolic blood pressure, mmHg	140 ± 20	148 ± 18	0.02
Diastolic blood pressure, mmHg	81 ± 12	76 ± 10	0.05
Haemoglobin, g/dL	12.3 ± 1.5	12.1 ± 1.4	0.30
Albumin, mmol/L	39 (38–42)	41 (37–43)	0.53
Cause of end stage renal disease, %			
-Diabetic nephropathy	15	0	0.02
-Nephrosclerosis	14	32	0.02
-Glomerulonephritis	33	21	0.52
-Inherited cystic kidney disease	10	18	0.28
-Other	27	29	0.82

**Fig. 2:**
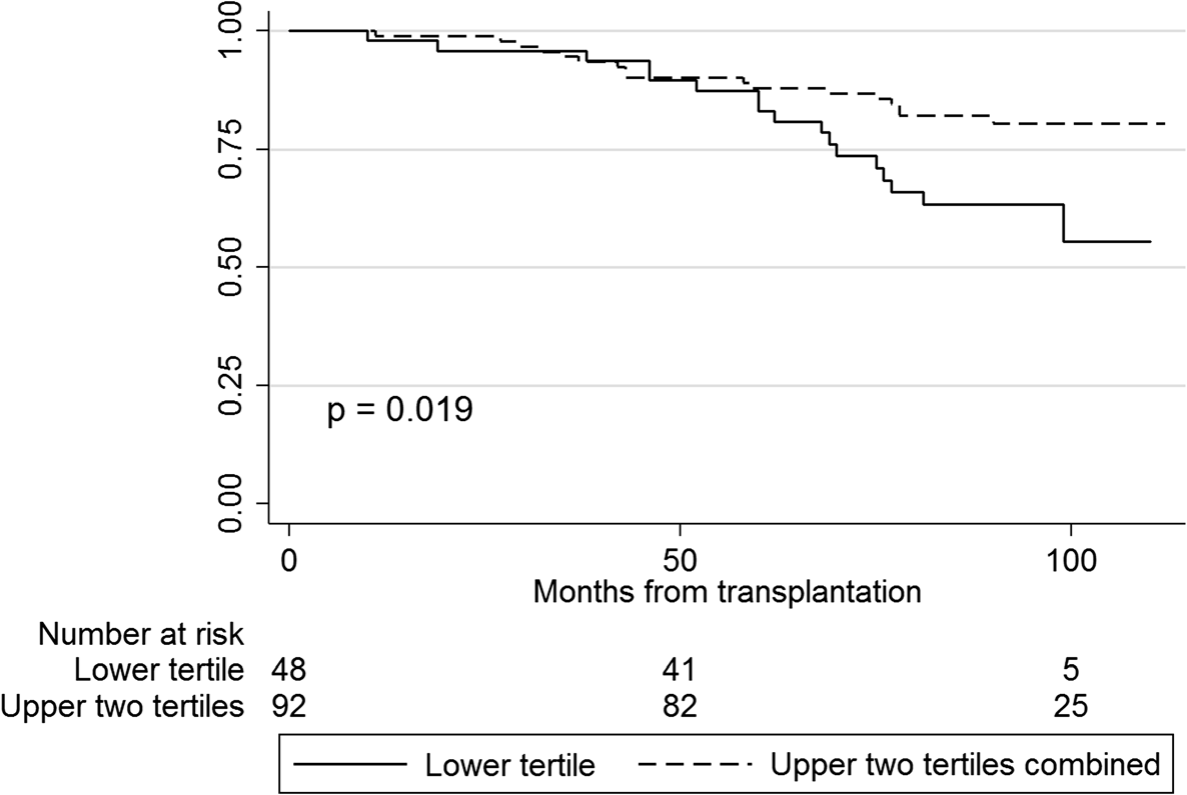
Kaplan-Meier plots showing survival of renal transplant patients with better vs. poorer scores in “physical function” obtained during dialysis

Differences in all-cause mortality after RTX appeared in the Kaplan-Meier survival plot between tertiles of patients with different scores of “physical function” measured in dialysis (*p* = 0.063). The two upper tertiles (with the best physical function) were combined as the curves were close and different from the curve of those with the worst perceived physical function (Fig. 2). Five years after RTX, 88 % of patients with good physical function (the upper two tertiles) were alive, and 83 % of patients with poor physical function (the lower tertile). No interaction between age and physical function was found (*p* = 0.38). Patient characteristics in the groups of poor vs. better physical function are given in Table 2. No other pretransplant KDQOL-SF domains were associated to survival after RTX.

**Table 2:** Characteristics of patients (*n* = 142) with different scores of “physical function” during dialysis

	Lower tertile physical function	Upper two tertiles physical function	*p* value
Age^a^	56 ± 14	50 ± 16	0.03
Female,%	34	32	0.64
Comorbidity^b^	5 (4–7)	4 (3–6)	0.02
Time in dialysis^a^	2.3 (1.5–3.5)	1.7 (1.1–2.5)	0.01
Albumin^c^	39.2 ± 4.4	39.6 ± 4.3	0.82
Living donor,%	17	32	0.06

Data presented as mean ± standard deviation or median with interquartile range as appropriate

^a^At time of renal transplantation, in years; ^b^Charlson Comorbidity Index in dialysis; ^c^In dialysis, mmol/l

### HRQOL in dialysis and graft function

Within the first year post-RTX two patients died, one of them experienced graft loss prior to death, and another three patients lost their graft within one year. These patients were not included in the regression analyses regarding GFR decline.

Mean GFR one year post-RTX was 60 ± 21 ml/min/1.73^2^, with a mean yearly eGFR decline of 2.4 ± 5.4 ml/min/1.73^2^. After adjustment for age, gender and comorbidity in a multivariate linear regression model, lower pretransplant score in the domain “mental health” was associated with worsened graft function, i.e. a larger yearly decline in eGFR (*p* = 0.048) (Table 3). None of the other generic or kidney-specific HRQOL domains were associated with graft function.

**Table 3:** Linear regression model showing associations of yearly decline in graft function in kidney transplanted patients (*n* = 137)

	Yearly GFR^a^ decline, ml/min/1.73 m^2^	
	B	p
Mental health^b^, +10 points	- 0.5	0.048
Age^c^,+10 years	- 0.3	0.29
Gender, female	0,3	0.47
Comorbidity^d^, +1 point	0.4	0.32

^a^Glomerular filtration rate. Higher values of yearly decline indicate more rapid loss of graft function from reference value one year after transplantation. ^b^Mental health measured in dialysis. ^c^Age at time of transplantation. ^d^Charlson comorbidity index without adding points for age

### Change in HRQOL from dialysis to transplantation and survival

Of the 142 transplanted patients, 110 participated in the follow-up study, providing data of change in HRQOL scores from dialysis to RTX. Baseline KDQOL-SF scores (in dialysis) of these patients and the proportion with clinical relevant change from dialysis to RTX are given in Table 4. Reasons for not taking part in the follow-up study were exclusion due to mental deterioration or prolonged hospitalization (*n* = 19), death before follow-up (*n* = 5), returned to dialysis or still in dialysis at time of follow-up (*n* = 5) and unwillingness (*n* = 3). The patients participating in the follow-up study did not differ in age, gender or comorbidity from those who did not take part, and the KDQOL-SF scores differed in two domains only, cognitive function (85 ± 17 vs. 74 ± 20, *p* = 0.001) and sexual function (71 ± 31 vs. 54 ± 33 *p* = 0.03) respectively.

**Table 4:** KDQOL-SF scores in dialysis and after transplantation (*n* = 110) and the proportion of patients with clinical relevant improvement^a^ from dialysis to transplantation^b^

	Scores in dialysis (mean ± SD)	Scores after transplantation (mean ± SD)	Proportion of patients with improvement^a^ %
Effect of kidney disease	69 ± 18	84 ± 16,	71
Burden of kidney disease	39 ± 26	73 ± 27	71
Symptoms	74 ± 16	82 ± 15	51
Work status	21 ± 35	45 ± 42	44
Sleep	61 ± 21	69 ± 20	35
Sexual function	71 ± 31	85 ± 20	25
Cognitive function	85 ± 17	88 ± 14	18
Social support	78 ± 28	83 ± 27	35
Quality of social interaction	82 ± 18	80 ± 18	23
Physical function	68 ± 24	74 ± 28	31
Role physical	36 ± 41	54 ± 44	44
General health	47 ± 22	59 ± 26	50
Vitality	46 ± 22	55 ± 24	48
Bodily pain	66 ± 27	73 ± 28	38
Social functioning	70 ± 29	81 ± 26	34
Mental health	77 ± 18	78 ± 19	26
Role emotional	65 ± 41	71 ± 42	27

^a^Improvement in score from dialysis to transplantation > 0.5 SD of the score in dialysis

^b^Scores previously presented as spider diagrams [9]

**Fig. 3:**
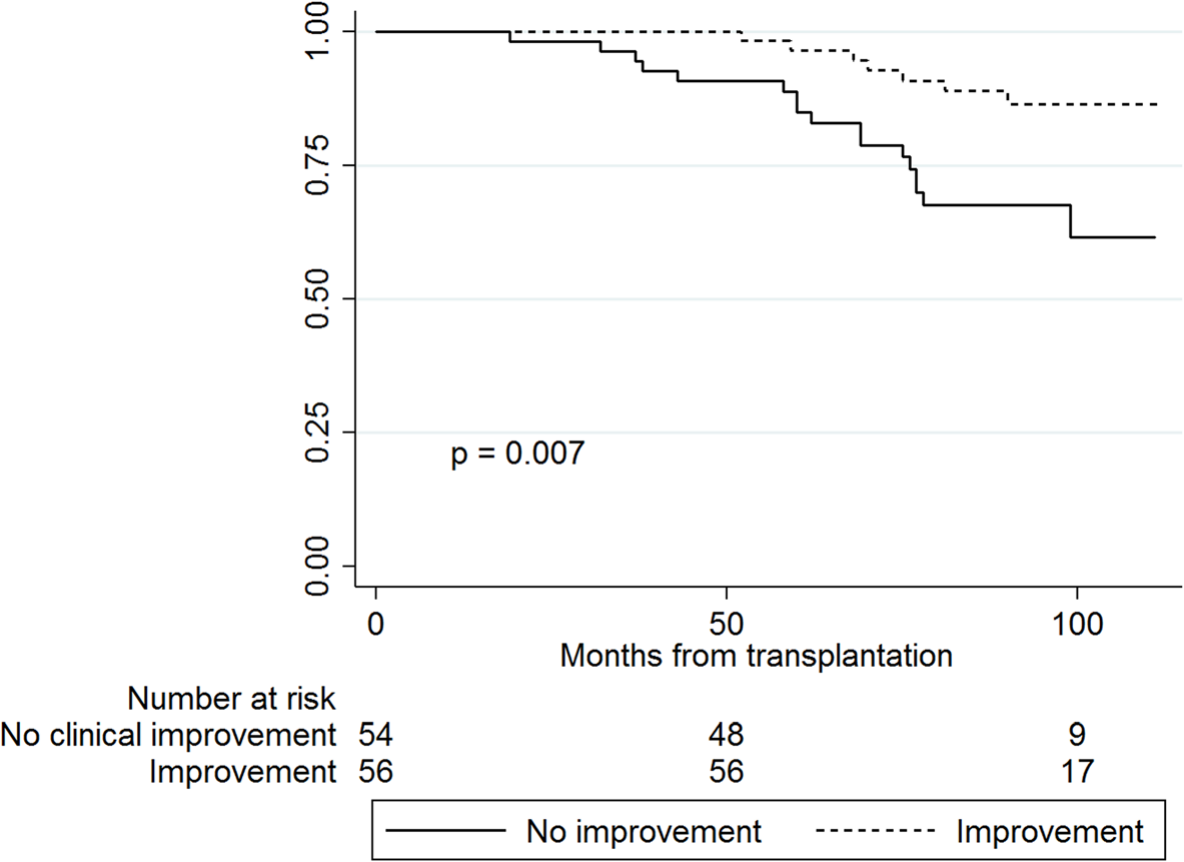
Kaplan-Meier plots showing survival of renal transplant patients with improvement vs. no improvement in “symptoms” from dialysis to transplantation

**Fig. 4:**
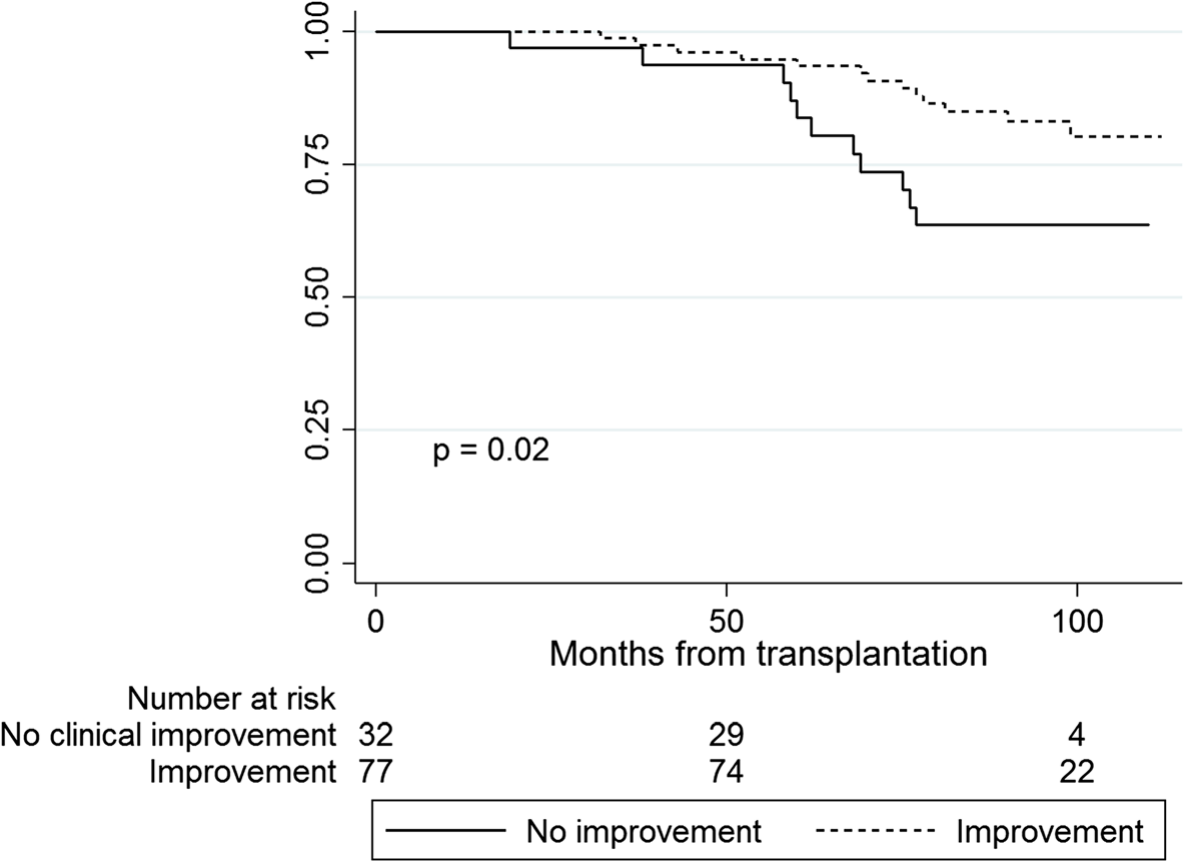
Kaplan-Meier plots showing survival of renal transplant patients with improvement vs. no improvement in “effect of kidney disease” from dialysis to transplantation

Kaplan-Meier curves revealed a survival advantage for patients with clinical relevant improvement from dialysis to transplantation in the HRQOL domains “symptoms” (*p* = 0.007) and “effect of kidney disease” (*p* = 0.02) compared to patients with no improvement or deterioration (Figs. 3 and 4). Five years after RTX, 96 % of the patients with improvement in symptoms were alive and 85 % of the patients with no improvement. For “effect of kidney disease”, the proportion of patients alive after five years was 94 % amongst those with improvement, and 84 % for patients with no improvement. Patient characteristics of the groups with and without clinical relevant improvement in symptoms and effect of kidney disease are given in Table 5. There was no statistical interaction between age and “symptoms” (*p* = 0.15) or between age and “effect of kidney disease” (*p* = 0.40) in the Cox regression survival analyses. Changes in the generic HRQOL (SF-36) domains from dialysis to transplantation did not predict survival

**Table 5:** Characteristics of patients (*n* = 110) with clinical relevant improvement vs. no improvement in the transition from dialysis to transplantation in the domains “symptoms” and “effect of kidney disease”

	Improvement in symptoms	No improvement in symptoms	*p* value	Improvement in effect of kidney disease	No improvement in effect of kidney disease	*p* value
Age^a^	48 ± 13	57 ± 16	<0.01	50 ± 15	57 ± 14	0.04
Female, %	32	35	0.74	27	47	0.05
Comorbidity^b^	4 (2–6)	5 (4–6)	0.02	4 (3–6)	5 (3–6)	0.53
Time in dialysis^a^	1.9 (1.0–2.5)	1.7 (1.3–3.3)	0.36	1.7 (1.1–2.7)	2.2 (1.4–3.7)	0.02
eGFR^c^	59 ± 21	56 ± 20	0.40	57 ± 19	61 ± 22	0.30
Living donor,%	26	32	0.47	32	19	0.27

Data presented as mean ± standard deviation or median with interquartile range as appropriate

^a^At time of renal transplantation, in years; ^b^Charlson Comorbidity Index in dialysis; ^c^Estimated glomerular filtration rate in ml/min/1.73^2^, at time of HRQOL measurement after transplantation

## Discussion

This prospective study is the first to address whether pretransplant generic and kidney-specific HRQOL domains measured in dialysis were associated with survival after renal transplantation. That a single domain, i.e. physical function, obtained during dialysis could serve as an indicator of survival after renal transplantation, is an important finding with possible clinical implications for the pretransplant evaluation.

Both kidney-specific and generic HRQOL domains have been found to be independent predictors of survival in dialysis patients, even after multiple adjustments, with physical function as the most powerful predictor [10, 12]. Physical aspects of HRQOL measured after transplantation are also independently associated to survival [13, 17]. Only two studies have previously addressed whether HRQOL measured during dialysis could affect survival after transplantation [14, 31]. Kutner et al. [14] reported that poor pretransplant physical function affected the combined endpoint hospitalization and death six years after transplantation. As the number of deaths was very low, only five, the endpoint seemed driven mainly by hospitalization. That death rate was much lower than what has been reported in other transplantation registries [32–34]. The inclusion criteria in that study may have favoured younger and possibly healthier patients with short time in dialysis before RTX. Furthermore, contrasting the present study, 71 % of the patients had been treated with peritoneal dialysis suggesting a certain selection of patients.

During the preparation of the present manuscript, a large retrospective registry study from the US was published, including 10875 patients subsequently transplanted [31]. Only the SF-36 domain “physical function” was investigated with regard to survival, and the authors concluded that poor functional status predicted 3-year mortality after transplantation. This is in accordance with our results from a much smaller cohort. In our prospective study, several other domains of generic and kidney-specific HRQOL were assessed with regard to survival. Thus, albeit small, the present study added new information, suggesting that the only KDQOL-SF domain measured in dialysis that was associated to survival after RTX, was the physical function. The large US registry study [15] corroborated our findings that the effect of physical function on survival was not affected by age, as no interaction was found. Johansen et al. [35] suggested that frailty in dialysis patients could be assessed using self-reported physical function (SF-36) rather than cumbersome objective testing. Frailty is a multidimensional construct describing vulnerability to various stressors [36]. The classic diagnostic criteria of frailty imbed both self-reported physical impairment and objective testing of muscle weakness. Frailty has been associated with disability, morbidity and mortality in older persons [36, 37]. Numerous dialysis patients suffer from severe neuropathy, myopathy and muscle weakness due to the kidney disease and possibly the dialysis treatment, explaining the high prevalence of frailty also in younger patients [38]. Comorbidity also contributes to frailty, and we observed that patients with better self-reported physical function had less comorbidity in the present study. That frailty observed in patients while in dialysis may affect mortality even after they have undergone renal transplantation, needs to be confirmed in larger, prospective studies.

If a single domain in KDQOL-SF, physical function, measured in dialysis may predict survival after RTX, this could be of clinical importance as it might serve to identify patients at increased risk after RTX. Furthermore, it may remind clinicians that physical activity in dialysis patients should be recommended as suggested in the Kidney Disease Outcomes Quality Initiative guidelines [39]. Self-reported physical activity has been shown to be associated with mortality and HRQOL both in dialysis patients and RTX patients [40, 41], but causality is not established and larger interventional studies are awaited. Yet another exciting and novel finding in the present study was that the domain mental health measured in dialysis was associated with graft function after RTX, even after multiple adjustments. Actually, in the regression model, poor mental health in dialysis was the only variable that predicted an accelerated loss of graft function. That pretransplant scores in mental health predicted decline in graft function has not previously been addressed. However, mental health as well as the mental composite score measured several years after transplantation has been shown to predict graft loss [13]. That effect on graft loss could not be confirmed in a later study of transplanted patients [17].

We have previously shown that there is surprisingly small changes in mental health from dialysis to transplantation [9]. Poor mental health is closely related to depressive symptoms [42, 43], which may increase the risk of non-adherence of immunosuppressive medication after transplantation [44]. Non-adherence in transplanted patients can be substantial [45], and enlarges the risk of graft failure [46]. Other factors contributing to accelerated graft decline in patients with poor mental health are likely numerous, including somatic, psychological, social and socioeconomic dimensions. Possible clinical implications of this finding should be closer follow-up of patients with low scores in mental health in dialysis.

We observed that patients with perceived clinical relevant improvement in the kidney specific domain “symptoms” and “effect of kidney disease” had a survival advantage compared to those without improvement. Comorbidity might have been a contributing factor. The domain “effect of kidney disease” encompasses questions about restrictions in everyday life due to the kidney disease. Improvement could reflect the perception of returning to “a normal life” after transplantation.

## Strengths and limitations

The prospective design, the representative cohort including a third of the prevalent dialysis population at the time of investigation, and that none of the patients was lost to follow-up, were important strengths of the study. The quality of the data was good, with less than 1.5 % missing values in SF-36. Data on renal function and mortality were complete. The acceptance criteria for renal transplantation were based on national consensus. All transplantations in Norway were performed in one national center.

A limitation of the study was the size of the cohort and the number of events. Due to the inclusion criteria, only clinically stable patients without prolonged hospitalization during the inclusion period, severe cognitive dysfunction, psychiatric illness or drug abuse were included. This may have led to selection bias towards healthier patients. As the majority of patients were Caucasians, the applicability to other populations may be limited. The transplantation rate in Norway is high, the time in dialysis is short, and the dialysis population in Norway may therefore differ from other dialysis populations with less access to renal transplantation [20]. Causality cannot be determined, as the study design is observational. The likelihood of type 1 errors increases when multiple comparisons are performed without statistical adjustments. This study is observational and with a limited sample size, hence no corrections were performed to avoid omitting of important clinical findings. Thus, the findings should be interpreted with caution.

## Conclusion

Poor physical function measured during dialysis was associated with increased mortality up to seven years after transplantation. This finding might have clinical implications. HRQOL measured in dialysis is a simple and time effective tool that could easily be included as a supplement to the pretransplant evaluation and particularly in recommendations with regard to physical function. Of clinical interest is also the finding that poor mental health in dialysis patients was associated to accelerated decline in graft function after RTX. Patients who perceive poor mental health in dialysis may need closer follow-up in the time after transplantation. Whether interventions to improve physical function and mental health in dialysis could affect survival even after transplantation needs to be confirmed in larger studies.

Improvement in HRQOL in the transition from dialysis to transplantation seemed to indicate improved survival. This new finding needs to be addressed in future studies.

## Abbreviations

CCI; Charlson Comorbidity Index; eGFR, Estimated glomerular filtration rate; EKD, Effect of kidney disease; ESRD: End stage renal disease; HD, hemodialysis; HRQOL, Health related quality of life; KDQOL, Kidney Disease Quality of Life; KDQOL-SF, Kidney Disease and Quality of Life Short Form measure version 1.3; PD, peritoneal dialysis; PROM, patient-related outcome measure; RTX, Renal transplantation; SF-36, Medical Outcome Study 36-item Short Form Health Survey.
